# Acknowledgment to the Reviewers of *Dentistry Journal* in 2022

**DOI:** 10.3390/dj11020029

**Published:** 2023-01-19

**Authors:** Dentistry Journal Editorial Office

**Affiliations:** MDPI AG, St. Alban-Anlage 66, 4052 Basel, Switzerland

High-quality academic publishing is built on rigorous peer review. *Dentistry Journal* able to uphold its high standards for published papers due to the outstanding efforts of our reviewers. Thanks to the efforts of our reviewers in 2022, the median time to first decision was 21 days and the median time to publication was 50 days. Regardless of whether the articles they examined were ultimately published, the editors would like to express their appreciation and thank the following reviewers for the time and dedication that they have shown *Dentistry Journal*:
Abdullah, Hasan ZuhudiLasota, AgnieszkaAbid, MushriqLassila, Lippo V. J.Achmad, HarunLeonardi, RosaliaAdamska, PaulinaLeón-Ríos, Ximena AlejandraAfrashtehfar, KelvinLeung, AlbertAgudelo-Suárez, Andrés AlonsoLi, PingAhmed, Muhammad AdeelLim, Mathew Albert Wei TingAhmed, NaseerLimsitthichaikoon, SucharatAlaeddini, MojganLin, Chin YiAlageel, OmarLin, WeichunAlagl, Adel S.Liu, HeAlam, Gazi MahabubulLiu, MengmengAlarcón, José AntonioLiu, Pearl PeiAl-Dwairi, ZiadLlena, Carmen C.Al-Haj Ali, Sanaa NajehLo Giudice, RobertoAl-Khalifa, Khalifa SulaimanŁobacz, MichałAlmas, Khalid M.Lombardi, TommasoAlmasan, OanaLoos, BrunoAl-Moraissi, Essam AhmedLopez-Jornet, PiaAlmotairy, NabeelLounis, MohamedAlves, RicardoLozano, AdriánAmara, Heithem BenLuchian, IonutAmo, Rocío OrtizLudovichetti, Francesco SaverioAndrukhov, OlehLung, Christie Ying KeiAngelopoulou, Matina V.Lupi, Saturnino MarcoAnić-Milošević, SandraMa, Kevin Sheng-KaiAnistoroaei, DanielaMadurantakam, Parthasarathy A.Antonelli, AlessandroMaeda, ShigeruAntoniadou, MariaMaftei, George AlexandruApostolova, ElisavetaMaglione, MicheleAra, ToshiakiMajeed, AbdulArbildo-Vega, HeberMalekigorji, MaryamArdelean, Lavinia CosminaMan, YiArdila, Carlos MartínMancini, LeonardoArnabat-Domínguez, JosepManso, Maria ConceiçãoArora, HimanshuMarini, LorenzoArregui, MaríaMarques Dos Santos, João MiguelAtsawasuwan, PhimonMarques, DuarteAttard, NikolaiMarrelli, MassimoAttia, SamehMartina, StefanoAudino, GiovanniMartínez-Beneyto, YolandaAuerkari, Elza IbrahimMartins, Jorge N. R.Babajko, SylvieMartín-Vacas, AndreaBabtan, Anida MariaMarto, Carlos MiguelBacci, ChristianMartu, IoanaBalcoş, CarinaMartu, SilviaBanerji, SubirMashima, IzumiBaráth, ZoltánMateos-Moreno, María VictoriaBarausse, CarloMatsuda, YuheiBarbato, LuigiMayta-Tovalino, FrankBardini, GiuliaMazur, MartaBarone, SeleneMelo, MaríaBecker, KathrinMelo, Mary AnneBencze, Maria-AngelicaMeneghello, RobertoBenito Sánchez, MaríaMesaros, Anca-StefaniaBennardo, FrancescoMeštrović, SenkaBensi, CaterinaMeurman, JukkaBenz, KorbinianMeyer, FredericBeretta, MarioMihan, NoalaBermúdez, MercedesMilic Lemic, AleksandraBernardi, SaraMisaki, TaroBersezio, CristianMishra, NeetaBerteşteanu, Şerban Vifor GabrielMitsea, AnastasiaBerton, FedericoMladenovic, RasaBettach, RaphaelMocny-Pachońska, KatarzynaBey, AfshanModha, BhavenBezerra-Neto, Jose. F.Mohannad, NassarBinmadi, NadaMonterubbianesi, RiccardoBjelica, RokoMoriyama, YasukoBobetsis, Yiorgos A.Mourao, CarlosBocchieri, SalvatoreMünchow, Eliseu AldrighiBociong, KingaMuniz, Francisco Wilker Mustafa GomesBottalico, LucreziaMuriel-Sánchez, Juan ManuelBottenberg, PeterMurmura, GiovannaBozhkova, TanyaMuronetz, VladimirBraz-Silva, Paulo HenriqueNagamatsu, YukiBrezulier, DamienNahajowski, MarekBrkić, HrvojeNajeeb, ShariqBruckmann, CorinnaNakashiro, Koh-IchiBrunello, GiuliaNakata, HidemiBud, MariusNalabothu, PrasadBurnheimer, John M.Nasiri, KavehBurns, LorelNeumann, Ana SolariButera, AndreaNgo, HienBylieva, DariaNica, DianaCampobasso, AlessandraNica, Luminiţa MariaCanciani, ElenaNicita, FabianaCantó-Navés, OriolNilius, ManfredCantore, StefaniaNishii, YasushiCapparè, PaoloNisi, MarcoCarossa, MassimoNovozhilova, NinaCarrard, Vinicius CoelhoNuzzolese, EmilioCarrilho, EuniceO’Connor-Reina, CarlosCarrillo-Díaz, MaríaOhe, Joo-YoungCaruso, SilviaOkada, YoshihiroCascone, PieroOkiji, TakashiCastroflorio, TommasoOkuyama, KoheiCattoni, FrancescaOliveros-López, Luis GuillermoChai, WenlinOlutola, Bukola G.Chalas, RenataOmran, MoustafaChang, Li-ChingOpris, HoriaChang, YuchaoOthman, AhmedChaturvedi, SaurabhOuchi, TakehitoChatzopoulos, Georgios SokratisPadilla, MarielaChauhan, PankajPagano, StefanoChaves-Neto, Antonio HernandesPałka, ŁukaszChebib, NajlaPalma, Paulo J.Chen, Chia-YuPalomo-Díez, SaraChen, YongPanda, SauravChen, Yo-WeiPaolone, GaetanoCheung, Gary Shun PanPaolone, Maria GiacintaChicos, Lucia-AntonetaPapazoglou, EfstratiosChisci, GlaucoPardal-Peláez, BeatrizChoi, Siu WaiParente, MarcoChrcanovic, BrunoPark, Jung-EunChu, Chun HungPascadopoli, MaurizioCichońska, DominikaPatel, MrudulaCoelho, AnaPatil, SantoshCofaru, Nicolae-FlorinPecci-Lloret, Miguel RamónCoro-Montanet, GleyvisPedullà, EugenioCorreia, MariaPelivan, IvicaCosta, Ana Luísa MoreiraPellegrini, Massimiliano MatteoCristalli, GiovanniPeng, Tzu-YuCruz-Hervert, Luis PabloPentapati, Kalyana ChakravarthyCurto, AdriánPerez, AlexandreD’anto, VincenzoPeřina, VojtěchD’Attilio, MichelePerrotti, VittoriaDa Cruz, MarianaPieniak, DanielDa Silva, Cláudia Helena LovatoPinchi, VilmaDalessandri, DomenicoPinho, TeresaDal-Fabbro, RenanPisani, FlavioDammaschke, TillPortelli, MarcoDe Almeida, Juliano MilaneziPorwal, AmitDe Angelis, NicolaPoulopoulos, AthanasiosDe La Garza-Ramos, Myriam AngélicaPreda, CamillaDe Mello Machado, Rafael CoutinhoPtasiewicz, MajaDe Nova-García, Manuel JoaquínPuranik, Chaitanya P.Degirmenci, AlperenPushalkar, SmrutiDekanic, AndreaQadir, SobanDellavia, ClaudiaQuinn, BarryDenis, FredericQuispe-López, NorbertoDerchi, GiacomoRadzki, DominikDevlin, HughRaghavan, SreevatsanDi Cosola, MicheleRamiro, Guillermo PradíesDi Nardo, DarioRashwan, MaherDi Venere, DanielaReda, RodolfoDing, XiuhuaReis, José AlexandreDivakar, DarshanRella, EdoardoDmello, CrismitaRibas Pérez, DavidDolić, OliveraRibeiro, Ricardo FariaDomínguez-Pérez, Rubén AbrahamRidwan, Rini DevijantiDritsas, KonstantinosRíos-Silva, MónicaDuanis-Assaf, DanielleRiviș, MirceaDumbryte, IrmaRobb, Nigel D.Duś-Ilnicka, Irena MałgorzataRober, AnthonyDyszkiewicz-Konwińska, MartaRoca-Millan, ElisabetEl Meligy, Omar Abd El SadekRoccuzzo, AndreaEl-Housseiny, AzzaRodakowska, EwaEliasz, WojciechRodrigues Pais Alves, Manuel FellipeEmam, MarwaRodríguez-Archilla, AlbertoEmodi-Perlman, AlonaRoggendorf, Matthias J.Ergün, GülfemRomeo, UmbertoEstrela, CarlosRubio Alonso, FaustoFahim, AyeshaRudolf, RebekaFakhruddin, Kausar SadiaRuggiero, GennaroFarronato, MarcoRuiz-Sánchez, CeliaFaruk, Simge TaşarRuksakiet, KasididFelippini, Ana Luiza De CarvalhoRungsiyakull, PimduenFernandes, GustavoRustico, LorenzoFernandes, JulianaSaadaldin, Selma AdnanFerreira, InêsSahrmann, PhilippFerrini, FrancescoSaito, KanFine, PeterSakai, HironoriFischer, NicholasSambataro, SergioFlapper, Walter J.Sánchez-Vargas, Luis OctavioFrança, RodrigoSantagostini, AntonioFrancisco, InêsSantilli, ManlioFranco, RoccoSantos, João M.Frankenberger, RolandSaran, StefanoFrascio, MattiaSarapultseva, MariaFráter, MárkSarul, MichałFrigo, LúcioSăveanu, Cătălina IuliaFukuhara, YokoSavignano, RobertoGabrić, DraganaSayed, MahmoudGad, Mohammed MoustafaSayed, MohammedGaffuri, FrancescaScardina, Giuseppe AlessandroGajera, MonaScholz, Konstantin JohannesGallo, SimoneSchüler, Ina ManuelaGándara-Vila, PilarSchulz, MatthiasGarcía-Pola, María JoséScolavino, SalvatoreGarcovich, DanieleSegù, MarziaGarus-Pakowska, AnnaSekiya, HidekiGasparro, RobertaŞentürk, Mehmet FatihGavic, LidiaSeracchiani, MarcoGavinha, SandraSerafin, MarcoGawda, PiotrSfeatcu, Ionela RuxandraGennai, StefanoShamel, MohamedGeorgaki, MariaSharafeldin, MohamedGeorgakopoulou, EleniSharma, DileepGerber, GáborSharpe, PaulGhasemi, ShohrehShiau, Harlan J.Gheorghe, Dorin-NicolaeShinya, AkikazuGherlone, EnricoShopova, DobromiraGibson, GretchenSignoriello, AnnaritaGibson, Monica PrasadSilveira, JoaoGijjapu, Durga RaoSimon, Brandenburg LeonardGlavina, DomagojSimon-Soro, AureaGlowacki, JakubSingaravel Chidambaranathan, AhilaGoldberg, Baruch R.Sinjari, BrunaGomez-Sosa, Jose FranciscoSlaidina, AndaGonzález-Aragón Pineda, Álvaro EdgarSmojver, IgorGórski, BartłomiejSoares, SimoneGrande, FrancescoSobkowiak, Michał J.Grocholewicz, KatarzynaSobral-Souza, Danielle FerreiraGrzech-Leśniak, KingaSoldatos, NikolaosGuazzo, RiccardoSolderer, AlexGuerrero-Gironés, JuliaSon, KeunBaDaGupta, AditiSong, Young WooHajeer, MohammadSoueidan, AssemHakeem, Faisal F.Spasojevic, Pavle M.Harrel, Stephen K.Stoetzer, MarcusHe, HongSufaru, Irina GeorgetaHe, PuhanSukegawa, ShintaroHedeșiu, MihaelaSukumaran, AnilHerrera-Atoche, José RubénSun, KuotingHnitecka, SylwiaSun, YingHong, BoyoungSurdacka, AnnaHosmani, JagadishSutej, IvanaHosseinpour, SepantaSuzuki, MarceloHowe, BrianSzabó, Bence TamásHsieh, Sung-ChihSzabó, GyörgyHuang, FumeiSzerszeń, MarcinHuang, YanSzymańska, JolantaHurjui, Loredana LilianaTaba Jr, MarioHuynh, NamTadin, AntonijaIdrees, MajdyTallarico, MarcoIlinca, RaduTano, RumiImbery, Terence A.Tatullo, MarcoImpellizzeri, AlessandraThurzo, AndrejInchingolo, FrancescoTian, KunInoue, GoTodaro, ClaudiaIonas, MonaTomas, MatejIshida, YoshikiTorres, JesúsIslam, Md. SofiqulTosco, VincenzoIzzetti, RossanaTraini, ToninoJanas-Naze, AnnaTribst, João Paulo MendesJang, JonghwaTroise, StefaniaJang, Jong-HwaTrybek, GrzegorzJaramillo, David E.Trzcionka, AgataJayasinghe, RuwanTsai, Ming-TzuJeavons, CharlotteTsoi, James Kit-honJedliński, MaciejTsolakis, Apostolos I.Jelovac, DragoÜnsal, GürkanJenkins, Susan J.Urbanowicz, MarcinJepsen, SörenUziel, NirJha, Saurav KumarVale, FranciscoJiang, Chloe MengValente, Nicola AlbertoJiang, GuoVan Den Boogaard, Marie-JoseJinno, YoheiVaz, PaulaJoachim, MichaelVégh, DánielJokić, Nataša IvančićVelasco Ortega, EugenioJorge, Paula KarineVernon, Jon J.Joshi, Vinayak M.Viciano, JoanJouhar, RizwanViegas, CarlosJumanca, DanielaVieira, Victor Talarico LealJunior, Sinval Adalberto RodriguesVilhena, Fabiano VieiraKallarakkal, ThomasVincent-Chong, Vui KingKämmerer, Peer W.Vinhas, Ana SofiaKarlović, ZoranVissink, ArjanKaruka, SiddarthVlachodimitropoulos, DimitriosKawase, TomoyukiVlasa, AlexandruKazazoglu, EnderVohra, FahimKern, WolfgangVuletić, MarkoKewalramani, NareshWalinski, Christopher J.Khacharem, AimenWalker, Alejandro R.Khaled, AhmedWalsh, Laurence J.Khan, HarisWang, HuiKhan, Ikram UllahWang, I-ChingKhan, Rabia SannamWide, UllaKharouf, NajiWójcik, DorotaKhiavi, Monir MoradzadehWong, May Chun MeiKim, Hae RanWong, RebeccaKim, Sung-KyoXhajanka, EditKlingberg, GunillaYamaguchi, MasaruKoka, SreenivasYamaguchi, SatoshiKomatsu, KeijiYamauti, MonicaKoppolu, PradeepYang, ShunfaKovačić, InesYavnai, NiritKozakiewicz, MarcinYazdanian, MohsenKranz, StefanYeh, Chih-KoKruse, TeresaYokose, SatoshiKsenia, BabinaYu, Ollie YiruKui, AndreeaYumoto, HiromichiKujan, OmarZadravec, DijanaKusumoto, JunyaZadrożny, ŁukaszKyaw, Thiha TinZafar, Muhammad SohailLabajo-González, ElenaZandinejad, AmiraliLaforgia, AlessandraZárate-Torres, Rodrigo ArturoLandi, LucaZsuzsanna, GurdánLanteri, Valentina



